# Clustering and classification for dry bean feature imbalanced data

**DOI:** 10.1038/s41598-024-82253-6

**Published:** 2024-12-28

**Authors:** Chou-Yuan Lee, Wei Wang, Jian-Qiong Huang

**Affiliations:** 1 School of Big Data, Fuzhou University of International Studies and Trade, Fuzhou, 350202 China; 2 School of Software, Yunnan University, Kunming, 650000 China

**Keywords:** K-means, BLSMOTE, Decision tree, Random forest, Support vector machine, Imbalanced data, Plant sciences, Mathematics and computing

## Abstract

The traditional machine learning methods such as decision tree (DT), random forest (RF), and support vector machine (SVM) have low classification performance. This paper proposes an algorithm for the dry bean dataset and obesity levels dataset that can balance the minority class and the majority class and has a clustering function to improve the traditional machine learning classification accuracy and various performance indicators such as precision, recall, f1-score, and area under curve (AUC) for imbalanced data. The key idea is to use the advantages of borderline-synthetic minority oversampling technique (BLSMOTE) to generate new samples using samples on the boundary of minority class samples to reduce the impact of noise on model building, and the advantages of K-means clustering to divide data into different groups according to similarities or common features. The results show that the proposed algorithm BLSMOTE + K-means + SVM is superior to other traditional machine learning methods in classification and various performance indicators. The BLSMOTE + K-means + DT generates decision rules for the dry bean dataset and the the obesity levels dataset, and the BLSMOTE + K-means + RF ranks the importance of explanatory variables. These experimental results can provide scientific evidence for decision-makers.

## Introduction

The dry beans are important food crops that can play an important role in addressing global food security and environmental challenges, while also contributing to healthy diets. The dry beans are an important and inexpensive source of plant protein, vitamins and minerals for people around the world. They are low in fat, cholesterol-free and an important source of dietary fiber. In addition, they are gluten-free and rich in minerals and B vitamins, which are important elements for a healthy life. With the rapid development of agricultural economic globalization, the economic exchange of dry beans among countries is becoming increasingly close. The dry beans are important edible beans for humans, they as a part of traditional diets have played an important role around the world. They are rich in nutrients and are a near perfect food that helps control weight, provide necessary nutrients for the body, and thus achieve disease prevention^1^. Although we have benefited greatly from dry beans, we must realize that as a common agricultural product, dry beans have a large market demand, and there is still a need to strengthen the information construction of various types of dry bean foods and promote economic development among trading countries. From an agricultural perspective, multiple cropping systems that include dry beans can increase soil fertility, improve yields and contribute to more sustainable food systems. Significantly, dry beans require much less water than other protein sources and can be grown in soils too poor to support other crops. The dry beans can also be used as animal feed, thus improving the quality of animal diets. In addition, dry beans can play an important role in climate change adaptation as they are rich in genetic diversity and can be used to breed climate-resilient varieties. If governments around the world increase their investment in the dry bean economy, it will surely promote the development of the dry bean industry. In view of the continuous growth of dry bean production, the increase in product varieties and the rapid development of the world economy, it is necessary to strengthen the research and analysis of dry beans, find potential factors to promote the development of the dry bean industry, strengthen dry bean production and promote technological innovation^2^.

The dry bean datasets are generally approached from both a data perspective and an algorithmic perspective, and a combination of both. For example, in 2022, Shahoveisi et al. used traditional machine learning methods to model the risk of disease development caused by sclerotinia sclerotiorum on rapeseed and dry beans^3^. In 2021, Mendigoria predicted the morphological characteristics and variety classification of dry beans through traditional machine learning methods^4^. However, the classification effect is not very good. This is because the dry bean dataset is an imbalanced data, which may cause the above scholars to have low classification accuracy and performance indicators such as precision, recall, f1-score, receiver operating characteristic (ROC) curve and area under the curve (AUC) in traditional machine learning methods such as decision tree (DT), random forest (RF), and support vector machine (SVM).

The imbalanced data refers to an imbalanced distribution of sample label values ​​in machine learning tasks. In classification problems, if the number of samples classified as negative (the majority class) far exceeds the number of samples classified as positive (the minority class), then the dataset can be considered imbalanced^5^. In many fields such as medicine, agriculture, and daily life, if minorities are ignored or misclassified, it will cause serious harm and negative impacts to individuals and society. The synthetic minority oversampling technique (SMOTE) is a method of randomly generating sample points based on sample distribution to reduce the imbalance of the dataset. Some scholars have used SMOTE to study sampling methods for imbalanced data. For example, in 2021, Wang et al. used SMOTE to expand and classify imbalanced data^6^. In 2022, Sun et al. et al. an ensemble model using K-means combined with SMOTE was used to predict stacking rockburst^7^. Although these scholars used SMOTE to process imbalanced data, the minority class samples on the borderline could not be processed, which easily caused the influence of noise during modeling, thus affecting the performance of data classification.

Since the dry bean dataset is an imbalanced data, this may lead to low classification accuracy and various performance indicators such as precision, recall, f1-score, ROC- AUC of traditional machine learning methods such DT, RF, and SVM. This study proposes a method different from the traditional machine learning method. It uses the borderline-synthetic minority class oversampling technique (BLSMOTE) and K-means combined with machine learning algorithms to predict dry bean varieties, that is, BLSMOTE + K-means + DT, BLSMOTE + K-means + RF, and BLSMOTE + K-means + SVM are proposed to improve the classification accuracy and various performance indicators such as precision, recall, f1-score, ROC-AUC of traditional machine learning methods such as DT, RF, and SVM. The main idea is to first use BLSMOTE to generate new samples for the samples on the boundary of the minority class samples of the dry bean dataset to reduce the impact of noise on model building, and then use K-means to cluster the dry bean data and divide the data into different clusters according to similarity or common features. The proposed algorithm BLSMOTE + K-means + SVM has better classification performance than other traditional machine learning methods, the BLSMOTE + K-means + DT provides decision rules for the dry bean dataset, and the BLSMOTE + K-means + RF is used to find the importance ranking of the factors affecting the dry bean features. In addition to the dry bean dataset, this study also used the obesity levels imbalanced dataset to test the performance of the proposed algorithm.

This paper collected University of California Irvine (UCI) dry beans dataset and shared by Selkuk University in Turkey, through R 4.3.2 software, and used BLSMOTE + K-means combined with machine learning methods, namely BLSMOTE + K-means + SVM, BLSMOTE + K-means + DT, and BLSMOTE + K-means + RF, to improve the classification performance such as classification accuracy, precision, recall, f1-score, and AUC of traditional machine algorithms, and also reflected the information closely related to dry bean characteristics and dry bean varieties. Furthermore, another obesity levels dataset is also used to test the performance of the proposed method.

The remainder of this paper is divided into four parts. Section 2 reviews the algorithms relevant to this study. The proposed method is presented in Sect. 3. Experimental results and discussions are presented in Sect. 4. Finally, Sect. 5 draws the conclusions of this study.

## Review of related algorithms

### Decision tree

The decision tree (DT) is a very important approach for providing decision-making in machine learning approaches. It is supervised learning, that is, the algorithm needs to be given certain samples. Each sample point instance has its own features and categories. Through these sample point information, the supervised learning algorithm can obtain the classification rules, and through the classification rules it can pass Features of new sample points to correctly classify them. Each leaf node corresponds to a decision, while the internal nodes and root nodes correspond to a test of a feature attribute; the samples contained in each node can be divided into sub-nodes based on the results of the attribute test; The complete set of samples is included in the root node of the decision tree, and the path from the root node to each leaf node corresponds to a set of attribute tests^8–10^. The information gain is an extremely important amount of data in the DT algorithm. Choosing the attribute with the highest information gain as the splitting attribute can promote the result partition to classify tuples with the smallest amount of information in the optional range, and the result is the most accurate. The calculation formula of information gain as shown in Eq. (1).

$$\:Info\left(D\right)=\left[-\sum\:_{i=1}^{m}{P}_{i}{{log}}_{2}\left({P}_{i}\right)\right]$$

$$\:{Info}_{A}\left(D\right)=\sum\:_{j=1}^{v}\frac{\left|{D}_{j}\right|}{\left|D\right|}\times\:Info\left(D\right)$$

1 $$Gain\: (A) =Info\:(D)-\:{Info}_{A}\left(D\right)$$

where $P_i$ represents the probability that the $i^{th}$ class appears in the entire training set, the Info (D) is the average amount of information needed to identify the class of a case in D, the $Info_A\;(D)$ is the expected information value for feature A to the partition D, the D is the number of cases in the training set, $D_j$ is a class, j = 1, 2, …, v and v is the number of classes, $D_j$ is a subset of D corresponding to the $j^{th}$ output, and $\mid{D}_{j}\mid$ is the number of cases of the subset $D_j$. It is necessary to set the complexity parameter (CP) and minimum split in DT to achieve a balance between accuracy and brevity.

### Random forest

The random forest (RF) is an algorithm that integrates multiple DT. It originates from the idea of classifier integration, which is to combine multiple classifiers to complete the classification task. Because the DT has poor generalization capabilities, the emergence of RF solves this problem. Because a decision tree has only one tree, its generalization ability is poor. Because random forest is composed of multiple DT, it has better generalization ability.

Selecting the best feature from the feature set of the current node is the rule for classifying features by the decision tree. However, in the random forest, the rule for classifying features is to select a single feature that contains m features for each node based on the decision tree. A subset of the node is randomly extracted from the feature set of the node, and then a single best feature is selected from the subset for partitioning^11–14^. The RF adopts a random approach in the feature selection process of each node, which randomly selects a portion of features from all features as candidate features, and then selects the optimal features for partitioning. In the prediction stage, each classification tree predicts new samples and generates a prediction result. The final prediction result is obtained through voting.

### Support vector machine

The support vector machine (SVM) is a machine learning approach that classifies a dataset into a hyperplane and solves the classification problem. The SVM solves the problem of Eq. (2) with a given training patterns $\:\left({x}_{i},\:{y}_{i}\right),\:i\:=\:1,\:2,\dots\:\:n,x\in\:{S}^{t},{y}_{i}\in\:\:\left\{-1,+1\right\}$, the feature input of a multi-dimensional feature vector of $\:{x}_{i}$ in the $\:{i}^{th}$ pattern, the number of patterns of $n$, the t-dimensional real number space of $S^t$, and the output of $y_i$. The SVM solves the problem shown in Eq. (2).

$$\text{Max}\;L(Q)\:=\sum\:_{i=1}^{n}{Q}_{i\:}-\:\frac{1}{2}\sum\:_{i,j=1}^{n}{Q}_{i}{Q}_{j}{y}_{i}{y}_{j}\langle{x}_{i}{,x}_{j}\rangle$$

2 $$\:s.t.\:\:0\le\:\:{Q}_{i}\:\le\:C,\:and\sum\:_{i=1}^{n}{Q}_{i}{y}_{i}=0$$

where $Q_i\;\geq$ 0 denotes the Lagrange multiplier and $C$ is a parameter of the cost of penalty. The feature space vectors $x_i,x_j$ are constructed in terms of the kernel $k$ where$k\left({x}_{i}{,x}_{j}\right)=\:\langle{x}_{i}{,x}_{j}\rangle.$ Using the feature space$k\left({x}_{i}{,x}_{j}\right)=\:\langle{x}_{i}{,x}_{j}\rangle$, the SVM can be expressed in Eq. (3).

3 $$\:{Max}\;L\left(Q\right)=\sum\:_{i=1}^{n}{Q}_{i\:}-\:\frac{1}{2}\sum\:_{i,j=1}^{n}{Q}_{i}{Q}_{j}{y}_{i}{y}_{j}k\left({x}_{i}{,x}_{j}\right)$$

For the radial basis function, it can be expressed as $\:k\left({x}_{i}{,x}_{j}\right)={exp}(-\gamma\:{\parallel{x}_{i}{-x}_{j}\parallel}^{2})$. Two parameters $C$ and $\gamma$ must be appropriately set in SVM. It is necessary to set $C$ and the $\gamma$parameters in the SVM to achieve a balance between accuracy and brevity^15–17^.

### BLSMOTE

The borderline-synthetic minority oversampling technique (BLSMOTE) is an oversampling method that is improved on the basis of synthetic minority oversampling technique (SMOTE) and uses samples on the boundary of minority class samples to generate new samples. The basic idea of ​​SMOTE is to generate new synthetic samples to balance the dataset based on the similarity between minority class samples. The basic principle is: first, calculate the Euclidean distance between the minority class sample and its neighboring samples, and then select the $\:k$ nearest neighbor samples; then, set the sample magnification to $\:M$ according to the sample ratio, randomly select a sample from the sample and name it $\:{X}_{i}$, $i=1,\:2,\:3,\:...,a$, and then randomly select a sample from its $\:k$ nearest neighbors and name it $\:{X}_{in}\:,\:\:n=\text{1,2},3,\dots\:,b$; finally, connect $\:{X}_{i}$ with $\:{X}_{in},$ and perform linear interpolation on the connecting line to generate a new artificial synthetic sample point $\:{X}_{newj}\:,\:\:j=1,\:2,\:3,\:...,t$. As shown in Eq. (4), the$\:\:rand\left(\text{0,1}\right)$ means to extract a random number between (0, 1), and $\:({X}_{in}-{X}_{i})$is the distance in the feature space^18^.

4 $$\:{X}_{newj}={X}_{i}+rand\left(\text{0,1}\right)\cdot\:\left({X}_{in}-{X}_{i}\right)$$

Because SMOTE is a method of randomly generating sample points based on sample distribution to reduce the imbalance of the dataset. It only considers a minority of samples and easily ignores the influence of surrounding samples. This can easily lead to the problem of repeated generated samples, thereby causing over-fitting. Compared with SMOTE, the BLSMOTE uses samples on the boundary of minority class samples to generate new samples, which can reduce the impact of noise on the model building.

### K-means

The K-means algorithm is a clustering algorithm. It is a representative of a typical objective function clustering approach. It uses a certain distance from a specific sample point in the sample space to the prototype as a reference for clustering, and uses functions to find the maximum and minimum values, and iteratively operates to finally obtain the clustering rules^19–21^.

For a certain sample in the sample space, calculate the Euclidean distance between sample points in different clusters and divide the sample space into $K$ clusters. Assuming that the clusters are divided into $C_1,\;C_2$, $C_k$, the expression of the Euclidean distance $E$ as Eq. (5)^22^.

5 $$\:E\:=\:\sum\:_{i=1}^{k}\sum\:_{x\in\:{C}_{i}}^{\:}{\parallel{x-u}_{i}\parallel}^{2}$$

The heuristic approach process of K-means is divided into the following steps: the first step is to select $K$ center points in the sample space; the second step is to calculate all the sample points in the sample space and calculate the distance to each center point. Euclidean distance, and classify each sample point and the nearest center point into the same category; the third step, recalculate the center point of each cluster in the sample space; the fourth step, based on the new center point, classify each sample in the sample space again; in the fifth step, repeat the third and fourth steps until the center point in the sample space no longer changes.

**Fig. 1:**
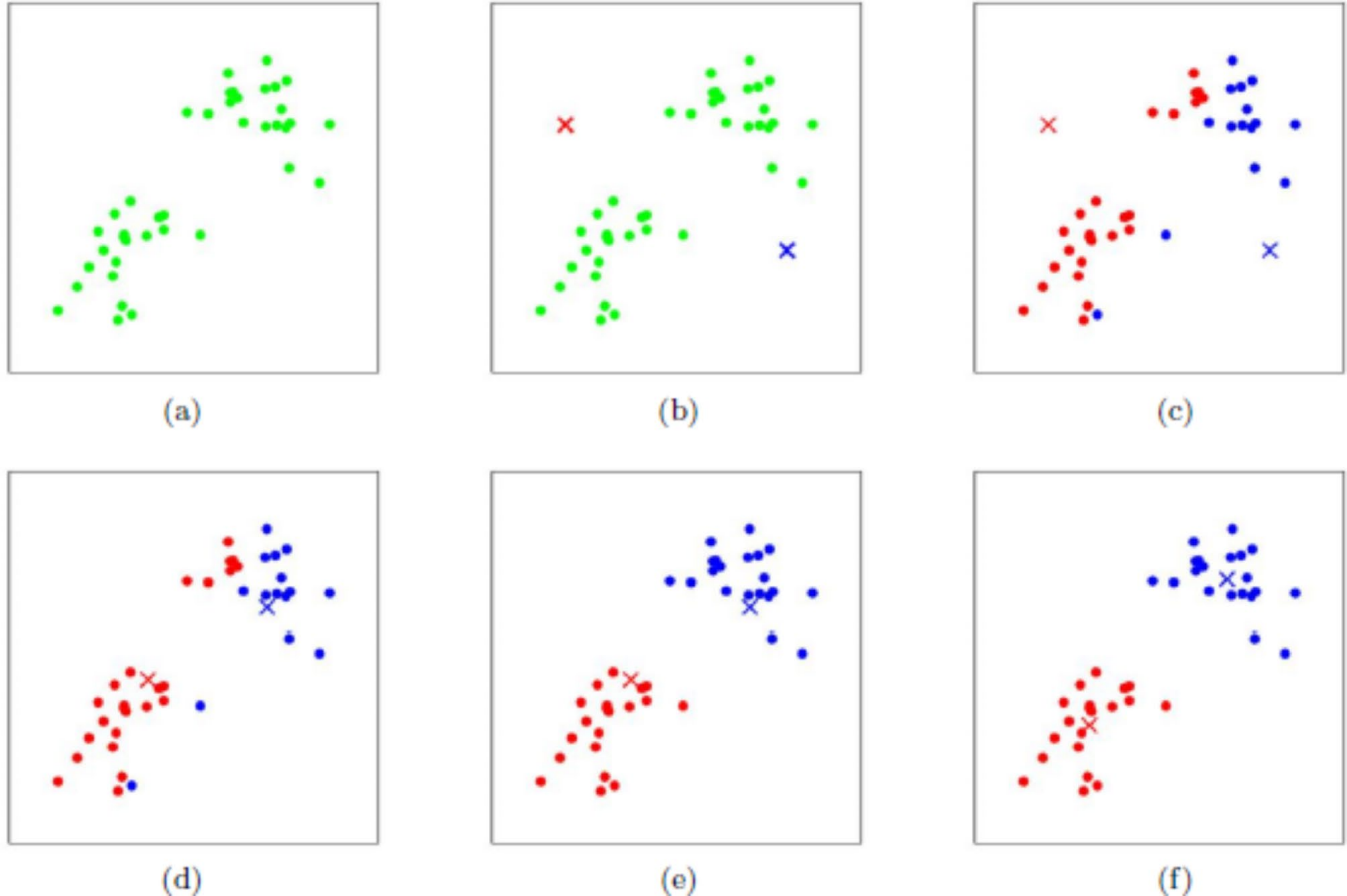
The process of K-means heuristic approach.

The process of the heuristic approach is shown in Fig. 1. All the samples in (a) select the initial center point to form (b). The distances between the sample points in (b) and the center point are calculated respectively, and further identified and classified. The cluster is formed (c), and then the center point is recalculated for each cluster to form (d). The above steps are repeated again to form a new cluster, which is formed (e). The (f) is formed when all center points no longer change.

##  Proposed methodology

### Data preprocessing

The dry bean dataset comes from the UCI dataset (http://archive.ics.uci.edu/ml/datasets/Dry+Bean+Dataset), which is collected and shared by Selkuk University in Turkey. This dataset has a total of 13,611 data, including 16 explanatory variables and 1 target variable. The 16 explanatory variables are Area, Perimeter, MajorAxisLength, MinorAxisLength, AspectRation, Eccentricity, ConvexArea, EquivDiameter, Extent, Solidity, Roundness, Compactness, ShapeFactor1, ShapeFactor2, ShapeFactor3, and ShapeFactor4. The dry bean dataset variables and their meanings are shown in Table 1.

**Table 1:** The dry bean dataset variable names and their meanings.

NO.	Name	Meanings
1	Area	The area of the dry bean region and the number of pixels within its boundaries
2	Perimeter	The dry bean border length
3	MajorAxisLength	The distance between the ends of the longest straight line that can be drawn in dry beans
4	MinorAxisLength	The longest distance that can be drawn when dry beans are perpendicular to the main axis
5	AspectRation	Define the relationship between MajorAxisLength and MinorAxisLength
6	Eccentricity	The ellipse has the same moment as the eccentricity of the region
7	ConvexArea	The number of pixels in the smallest convex polygon that can contain a dry bean.
8	EquivDiameter	The diameter of a circle equal to the area of a dry bean.
9	Extent	The ratio of pixels in the dry bean bounding box to the dry bean area
10	Solidity	The ratio of pixels in the convex shell of dry beans to the pixels in dry beans
11	Roundness	$\frac{4\cdot\pi\:\cdot\text{Area}}{\text{Perimeter}^{2}}$
12	Compactness	$\frac{\text{EquivDiameter}}{\text{MajorAxisLength}}$
13	ShapeFactor1	$\:\frac{\text{MajorAxisLength}}{Area}$
14	ShapeFactor2	$\:\frac{\text{MinorAxisLength}}{Area}$
15	ShapeFactor3	$\:\frac{4\cdot\text{Area}}{{{\uppi\:}\cdot\text{MajorAxisLength}}^{2}}$
16	ShapeFactor4	$\:\frac{4\cdot{Area}}{\pi\:\cdot\text{MajorAxisLength}\cdot\text{MinorAxisLength}}$
17	Class	The target variable has seven categories: Seker, Barbunya, Bombay, Cali, Dermosan, Horoz and Sira.

**Fig. 2:**
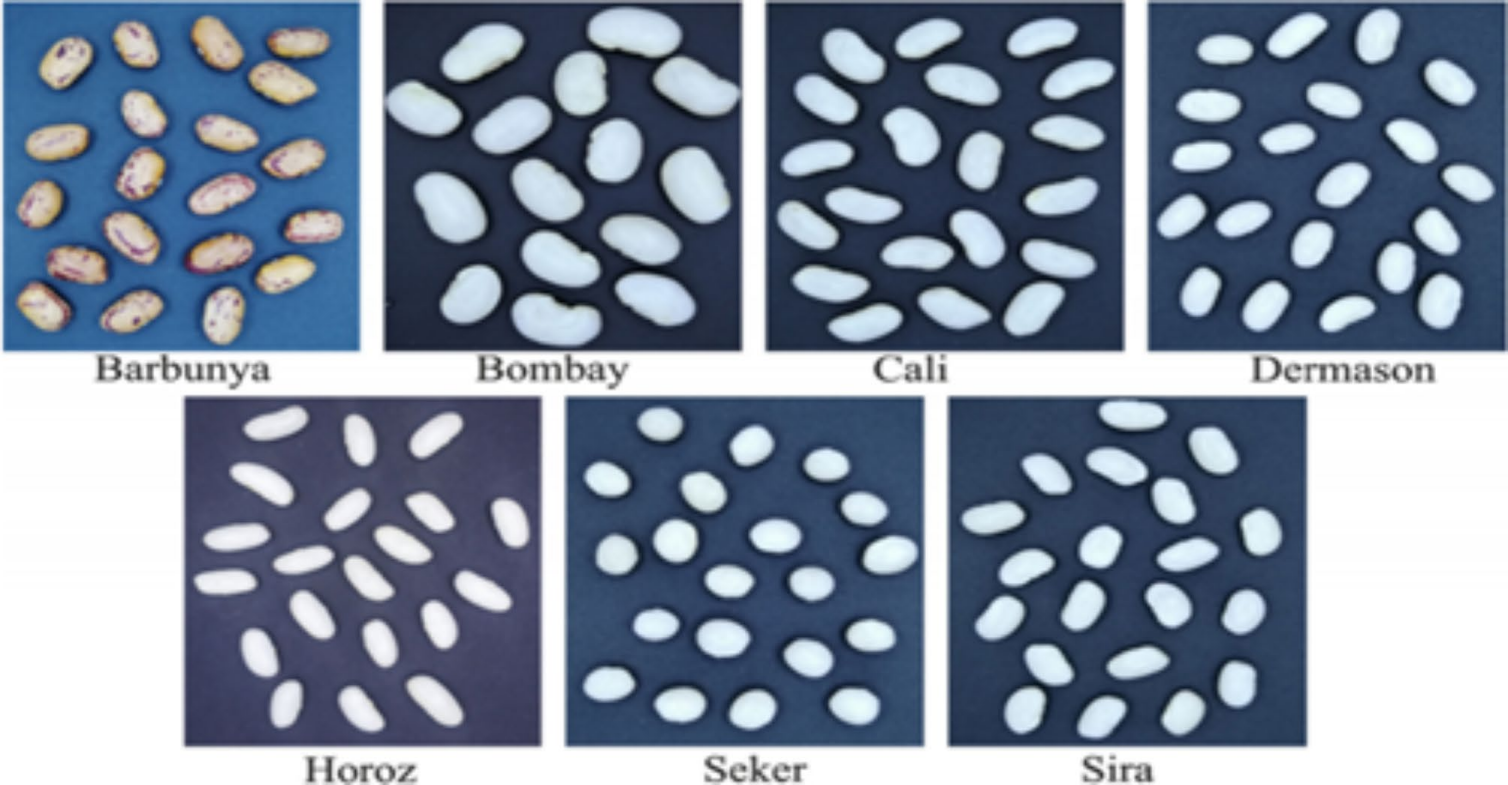
The category 1–7 sampling diagram of the target variable.

The target variable of this dataset has seven different types of dry beans, taking into account their morphology, shape, type, structure and other features, namely Barbunya, Bombay, Cali, Dermason, Horoz, Seker and Sira. In the classification model, a high-resolution camera was used to image 13,611 grains of 7 different types of dry beans. Figure 2is a sampling diagram of categories 1–7 of the target variable^23,24^.

Furthermore, the obesity levels dataset collected from UCI repository (https://archive.ics.uci.edu/dataset/544/estimation+of+obesity+levels+based+on+eating+habits+and+physical+condition is also used to test the performance for the proposed method. The obesity levels dataset contains 2111 records, consisting of 16 explanatory variables and 1 target variable. The target variable consists of seven different obesity levels, including Insufficiency weight, normal weight, overweight level I, overweight level II, obesity type I, obesity type II, and obesity type III. The obesity levels dataset variables and their meanings are shown in Table 2.

**Table 2:** The obesity levels dataset variable names and their meanings.

No.	name	Meanings
1	Gender	Male and female
2	Age	Age, taken between 14 and 61 years old
3	Height	Participants’ height (meters)
4	Weight	Participant’s weight (kg)
5	Family_history_with_over	Are family members overweight?
6	FAVC	Do you often eat high calorie foods?
7	FCVC	Will you eat vegeles during meals?
8	NCP	How many main meals do you eat every day?
9	CAEC	The frequency of eating food between meals
10	SMOKE	Smoking Status
11	CH2O	How much water do you drink every day?
12	SCC	Are calories monitored daily?
13	FAF	How often do I engage in physical activity?
14	TUE	What is the time spent using technology devices such as mobile phones?
15	CALC	How often do you drink alcohol?
16	MTRANS	What kind of transportation is usually used?
17	NObeyesdad	The target variable has seven grades such as insufficient_weight, normal_weight, overweight_level_I, overweight_level_II, obesity_type_I, obesity_type_II, and obesity_type_III

### Research process

The dry bean dataset in this paper comes from UCI. In order to facilitate prediction, the value of the target variable of the dataset is converted from a string to a number. The data is divided into ten parts. Eight parts of the data are retrieved as training data and the other two parts are used as test data, and all comparisons are made under fair conditions.

**Fig. 3:**
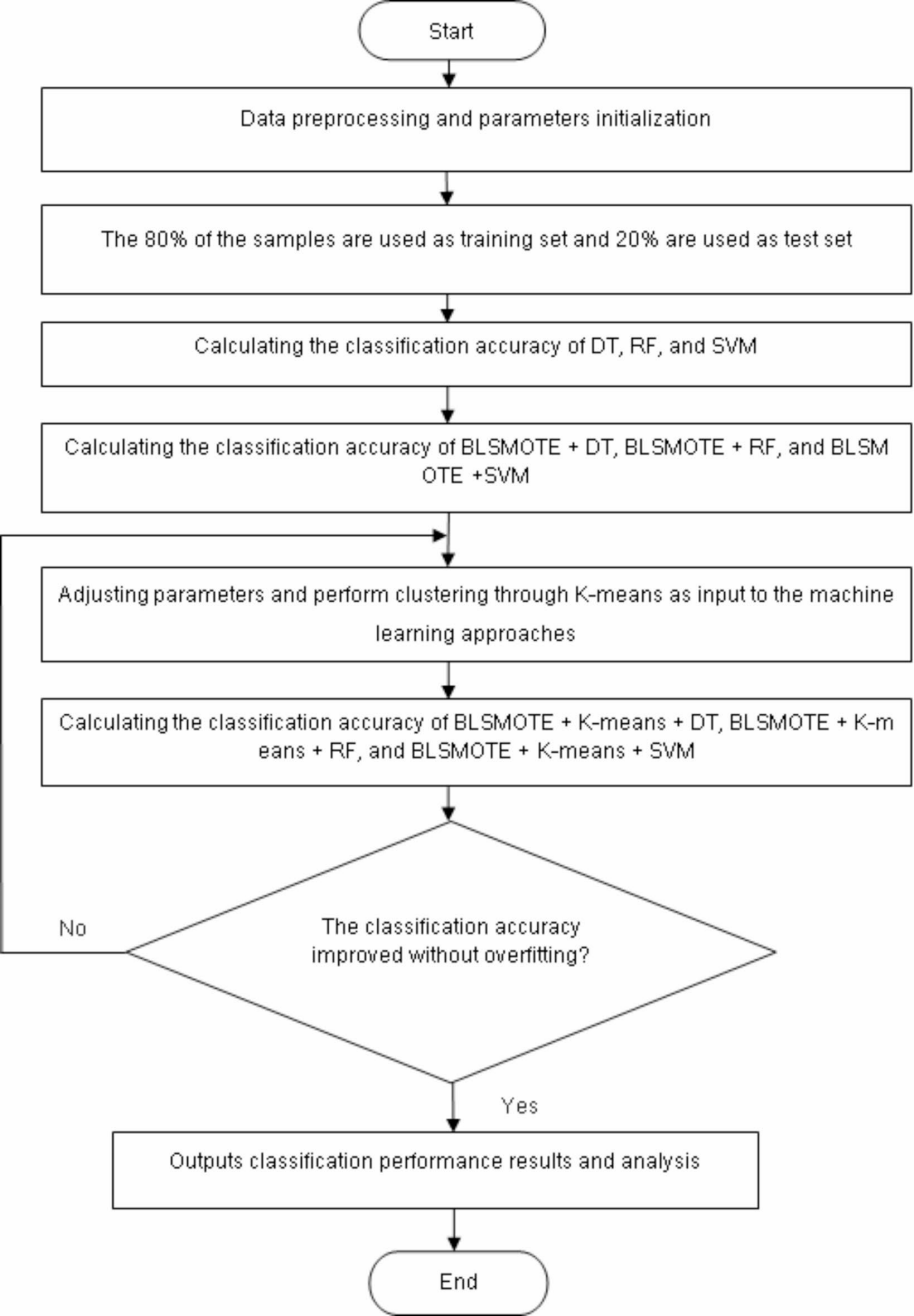
The flow chart of BLSMOTE + K-means + machine learning approaches.

Since the dry bean dataset is an imbalanced data, this may lead to low classification accuracy and various performance indicators such as precision, recall, f1-score, ROC-AUC of traditional machine learning methods such as DT, RF, and SVM. In order to improve this shortcoming, this paper proposes a BLSMOTE and K-means algorithm combined with DT, RF and SVM, namely BLSMOTE + K-means + DT, BLSMOTE + K-means + RF, and BLSMOTE + K-means + SVM for clustering and classification of dry bean characteristics. In the proposed algorithm, BLSMOTE generates new samples for samples on the boundary of minority class samples in the dry bean dataset to reduce the impact of noise on model building, and K-means clusters the dry bean data and divides the data into different clusters according to similarity or common features. The flowchart of the proposed algorithm is shown in Fig. 3. In Fig. 3, after preprocessing the dry bean dataset, the Kvalue of K-means is parameterized, and the parameter of DT, RF, and SVM are set. After that, calculating the classification accuracy of DT, RF, and SVM, and then the classification accuracy of BLSMOTE + DT, BLSMOTE + RF, and BLSMOTE + SVM are calculated, and then the classification accuracy of BLSMOTE + K-means + DT, BLSMOTE + K-means + RF, and BLSMOTE + K-means + SVM are calculated, and the parameters were adjusted to improve the training set classification accuracy and test set classification accuracy without over-fitting^17^. Finally output the classification accuracy and analyze the results.

## Experimental results and discussions

### Data source and preprocessing

The dry bean dataset collected by UCI provides information on 13,611 dry beans (target variable has seven different varieties) from Selkuk University in Turkey. This study uses digital transformation to convert the value of the target variable from a string to a numeric value so that the subsequent machine learning algorithm can classify the dry bean dataset. The target variable content, such as Seker, Barbunya, Bombay, Cali, Horoz, Sira, and Dermason, are replaced with 1 to 7 respectively.

Furthermore, the textual content of the obesity levels dataset of 2,111 records was replaced with numerical content using digital conversion. The specific method is to convert the contents of the explanatory variables (CAEC) and (CALC) such as no, sometimes, frequently, and always to 1, 2, 3, and 4 respectively. Replace the contents of the explanatory variables (MTRANS) such as bike, motorbike, walking, automobile, and publicTransportation with 1, 2, 3, 4, and 5 respectively. Represent the contents of the explanation variables (family_history_with_overweight), (FAVC), (SMOKE), and (SCC) with 0 for no and 1 for yes. The content of Gender is represented by 0 for female and 1 for male. Replace the contents of the target variable such as insufficient_weight, normal_weight, overweight_level_I, overweight_level_II, obesity_type_I, obesity_type_II, and obesity_type_III with 0 to 6, respectively.

### The calculation of classification accuracy and other performance metrics

The confusion matrix is a standard form of presenting accuracy estimates in matrix form. This paper uses a confusion matrix to calculate the classification accuracy of the algorithm results. The confusion matrix is shown in Table 3.

**Table 3:** The confusion matrix.

	Predicted positive	Predicted negative
Actual Positive	TP (true positive)	FN (false negative)
Actual Negative	FP (false positive)	TN (true negative)

In Table 3, the TP and TN represent the results of correct prediction. The FP and FN represent the results of incorrect prediction. The classification accuracy is calculated as shown in Eq. (6):.

6 $$\:Classification\:accuracy=\frac{TN+TP}{TN+TP+FN+FP}\cdot\:100{\%}$$

The precision is proposed based on the prediction results, representing the correct probability of the classification model predicting a positive result. The calculation method is shown in Eq. (7).

7 $$\:Precision=\frac{TP}{FP+TP}$$

The recall represents the probability of being successfully predicted when the event we are concerned about actually occurs, calculated using Eq. (8).

8 $$\:Recall=\frac{TP}{FN+TP}$$

The f1-score is a comprehensive evaluation index that combines precision and recall, and is the harmonic mean of the two^25–27^. The calculation method is shown in Eq. (9).

9 $$\:F1-score=\frac{2\cdot\:Precision\cdot\:Recall}{Precision+Recall}$$

The AUC value is expressed as the area under the ROC curve. The AUC ranges from 0 to 1. A higher value indicates better classifier performance. The ROC curve shows the relationship between false positive rate (FPR) and true positive rate (TPR). Among them, the horizontal axis is FPR, which represents the proportion of samples that are incorrectly predicted as positive categories in the actual negative categories.The calculation is shown in Eq. (10). The vertical axis is TPR, which represents the proportion of correctly predicted positive categories in the actual positive category samples. The calculation is shown in Eq. (11).

10 $$\:TPR=\frac{TP}{TP+FN}$$

11 $$\:FPR=\frac{FP}{FP+TN}$$

### Comparative analysis of BLSMOTE + K-means + SVM and other methods

The cross-validation is a very important technique in training models, which can avoid overfitting of models. It provides us with a more accurate way to estimate the prediction performance of models, and can also improve the generalization ability of models. In order to fairly compare the prediction accuracy of various algorithms, each method in this study utilized 10-fold cross-validation to calculate the prediction accuracy. The data is divided into 10 parts. Eight parts of the data are retrieved as training data and the other two parts are used as test data. The complexity parameter (CP) of DT is set to 0.005, and the minimum number of branch nodes (Minsplit) is set to 10^28^. The number of trees in RF is set to 100. The settings of C and parameters will affect the eslishment of the support vector machine model. This article sets the C value to 1 and the value to 0.4^29^. The K value of K-means is set to 7, which means it is divided into 7 clusters. To illustrate the effectiveness of the proposed algorithm, the experimental results are shown from Tables 4 and 5.

**Table 4:** The classification accuracy and performance indicators of dry bean dataset.

Approaches	Training set accuracy (%)	Test set accuracy (%)	Precision	Recall	F1-score	AUC
DT	89.20	88.83	0.9042	0.8959	0.9000	0.9088
BLSMOTE + DT	89.25	89.03	0.9071	0.8973	0.9022	0.9016
K-means + DT	89.29	88.61	0.9076	0.8989	0.9032	0.9085
BLSMOTE + K-means + DT	89.98	89.81	0.9098	0.9069	0.9083	0.9102
RF	92.46	83.25	0.9053	0.9071	0.9062	0.9245
BLSMOTE + RF	92.51	84.95	0.9033	0.9031	0.9032	0.9259
K-means + RF	92.57	84.31	0.9073	0.9023	0.9048	0.9266
BLSMOTE + K-means + RF	92.58	86.06	0.9001	0.9183	0.9091	0.9277
SVM	94.01	93.75	0.9402	0.9437	0.9419	0.9527
BLSMOTE + SVM	94.25	93.09	0.9336	0.9395	0.9365	0.9541
K-means + SVM	98.79	96.98	0.9644	0.9691	0.9667	0.9568
BLSMOTE + K-means + SVM	98.86	97.54	0.9736	0.9743	0.9739	0.9831

**Table 5:** The classification accuracy and performance indicators of obesity levels dataset.

Approaches	Training set accuracy	Test set accuracy (%)	Precision	Recall	F1-score	AUC
DT	90.41	89.60	0.8052	0.8151	0.8101	0.9312
BLSMOTE + DT	90.88	89.03	0.8055	0.8158	0.8106	0.9416
K-means + DT	91.56	89.07	0.8193	0.8154	0.8173	0.9635
BLSMOTE + K-means + DT	93.29	90.07	0.8060	0.8765	0.8398	0.9642
RF	97.99	90.20	0.8668	0.8754	0.8711	0.9747
BLSMOTE + RF	98.51	90.95	0.8751	0.8763	0.8757	0.9772
K-means + RF	99.01	91.25	0.8713	0.8852	0.8782	0.9833
BLSMOTE + K-means + RF	99.13	91.49	0.9046	0.9256	0.9150	0.9835
SVM	98.99	92.62	0.9402	0.9437	0.9419	0.9807
BLSMOTE + SVM	99.16	93.09	0.9436	0.9495	0.9465	0.9841
K-means + SVM	99.31	93.58	0.9644	0.9691	0.9667	0.9868
BLSMOTE + K-means + SVM	99.57	94.09	0.9736	0.9743	0.9739	0.9885

**Fig. 4:**
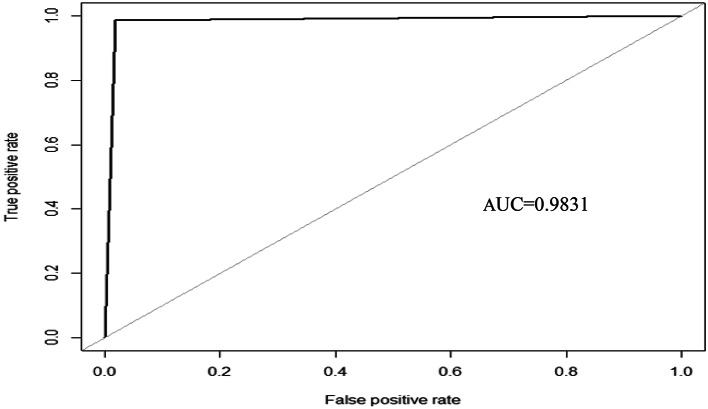
The ROC curve and AUC value of BLSMOTE + K-means + SVM for dry bean dataset.

**Fig. 5:**
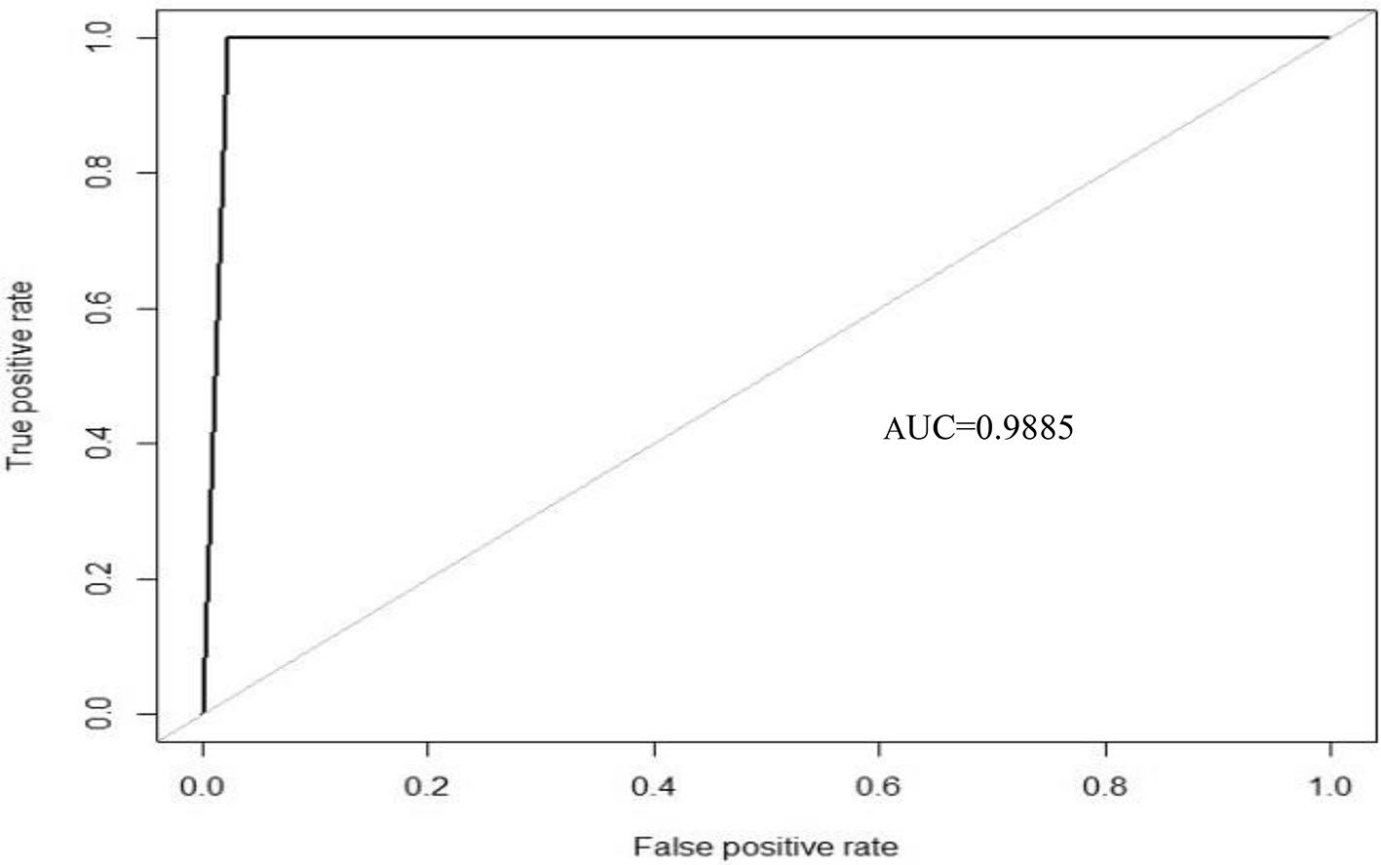
The ROC curve and AUC value of BLSMOTE + K-means + SVM for obesity levels dataset.

1. The comparison results in Table 4 show that the classification accuracy of the BLSMOTE + K-means + SVM training set in the dry bean dataset is 98.86%, which is better than the 94.01% of only SVM. Also from Table 5, we can find that the classification accuracy of the training set of the BLSMOTE + K-means + SVM on the obesity levels dataset is 99.57%, which is better than the 98.99% of only SVM. Because the imbalanced data is processed by BLSMOTE, it has the advantage of using samples on the boundary of minority class samples to generate new samples, which can reduce the impact of noise on model building, and K-means clustering has the advantage of dividing data into different groups according to similarity or common features, so BLSMOTE + K-means + SVM has better classification performance than only SVM in Tables 4 and 5.
2. The comparison results in Table 4 show that the classification accuracy of BLSMOTE + K-means + SVM on the dry bean dataset on the training set is 98.86%, which is better than 92.58% of BLSMOTE + K-means + RF and 89.98% of BLSMOTE + K-means + DT. Table 5 shows the classification accuracy of BLSMOTE + K-means + SVM on the obesity levels dataset on the training set is 99.57%, which is better than 99.13% of BLSMOTE + K-means + RF and 93.29% of BLSMOTE + K-means + DT. Since SVM is a hyperplane classifier in BLSMOTE + K-means + SVM, while RF and DT are classifiers based on tree structures in BLSMOTE + K-means + RF and BLSMOTE + K-means + DT. The BLSMOTE + K-means + SVM has the advantage of hyperplane classification, so its performance is better than the tree classification of BLSMOTE + K-means + RF and BLSMOTE + K-means + DT in Tables 4 and 5.
3. Comparing the results in Table 4, in addition to the classification accuracy of BLSMOTE + K-means + SVM of the dry bean dataset, the performance indicators of precision, recall, f1-score, and AUC are relatively the best compared to other algorithms, with an AUC of 0.9831, and its ROC-AUC is shown in Fig. 4. Comparing the results in Table 5, the AUC of BLSMOTE + K-means + SVM for the obesity levels dataset is 0.9885, and its ROC- AUC is shown in Fig. 5.

### The analysis and discussion of K-means clustering

**Fig. 6:**
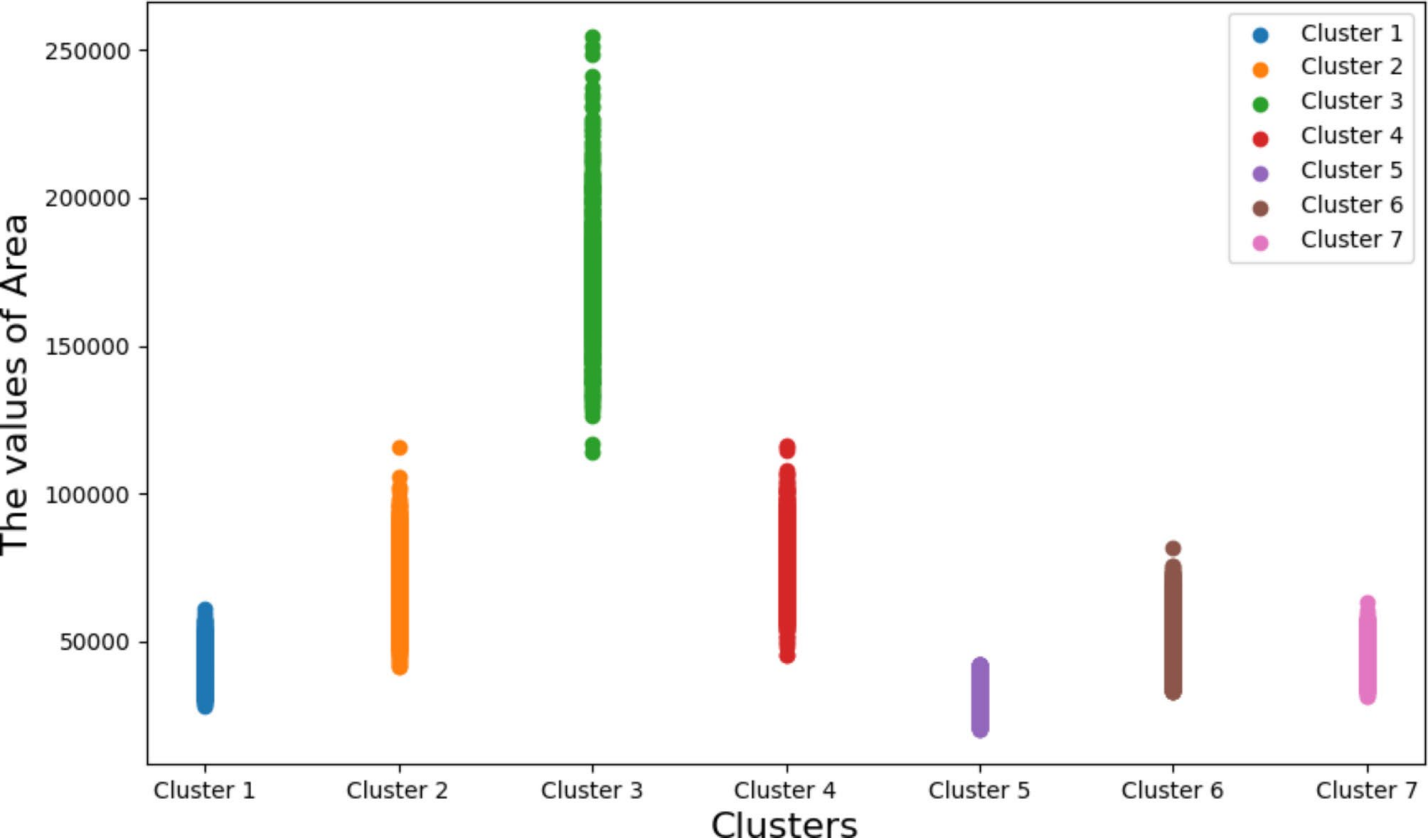
The dry bean data is divided into 7 clusters using feature (Area) through K-means.

After the dry bean data is balanced by BLSMOTE, the K-means is used for clustering, which can divide the data into different groups based on similarities or common features. Among the 16 explanatory variables of dry beans, this feature (Area) and other explanatory variables such as Roundness, ShapeFactor1, ShapeFactor2, ShapeFactor3, and ShapeFactor4 has a close relationship, so the feature (Area) is used to illustrate the seven clusters divided by K-means, as shown in Fig. 6. In Fig. 6, the horizontal axis is the seven clusters divided by K-means, and the vertical axis is the value of (Area). It can be found that the maximum value in the feature (Area) falls in the cluster 3, and the minimum value in the feature (Area) falls in the cluster 5.

**Fig. 7:**
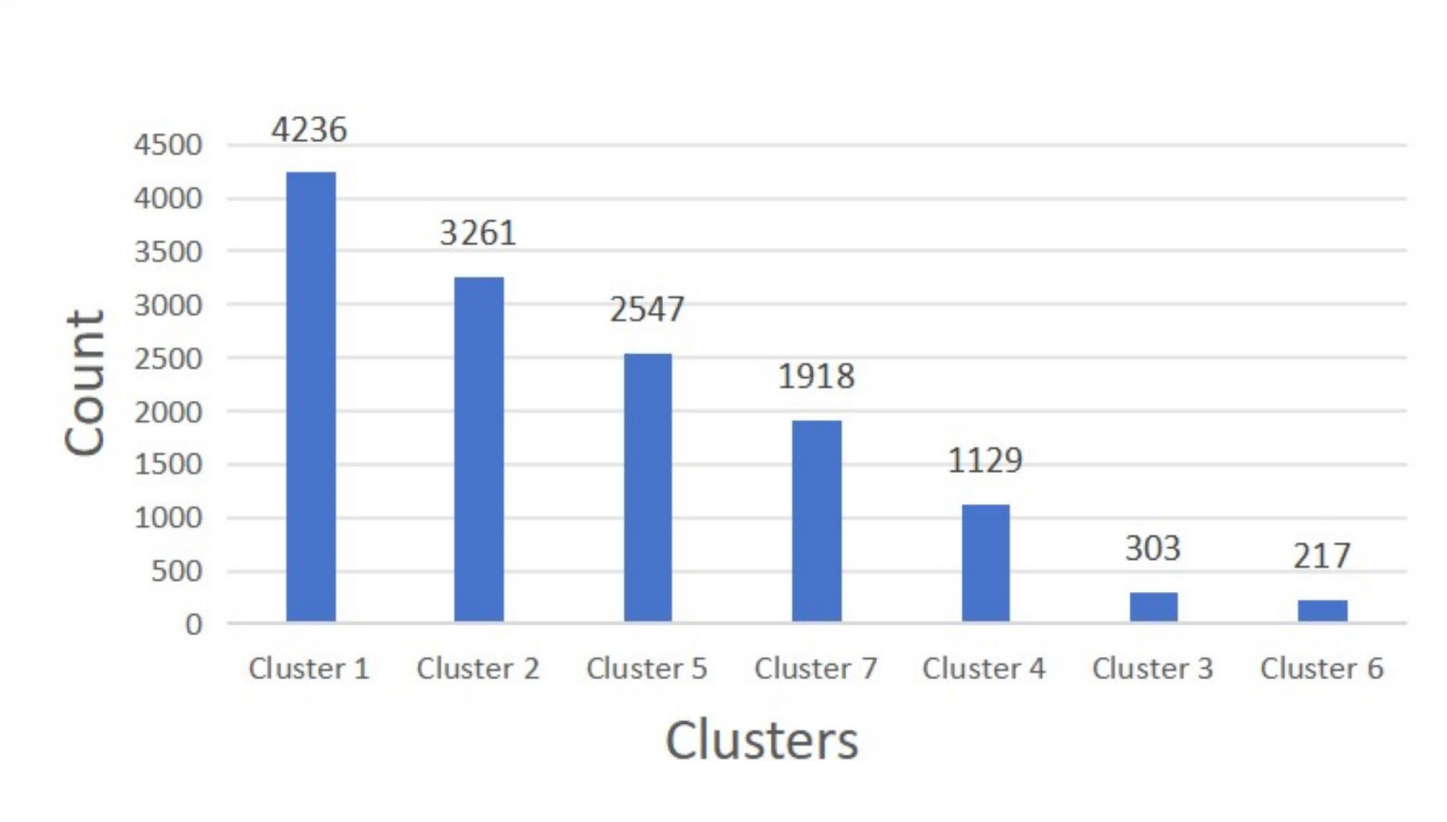
The clustering diagram of dry bean dataset.

The dry bean dataset has a total of 13,611 data. After K-means clustering, seven clusters are formed. The number of records in each cluster is presented from most to least, as shown in Fig. 7. From Fig. 7, we can find the cluster 1 has 4,236 records, which is the largest number of records among the seven clusters, which means that features with more similarities or commonalities are drawn in the cluster 1. The cluster 2 has 3,261 records, the cluster 5 has 2,547 records, the cluster 7 has 1,918 records, the cluster 4 has 1,129 records, the cluster 3 has 303 records, and the cluster 6 has 217 records, which is the least number of records among the seven clusters, which means that the features with the least similarity or commonality are plotted in cluster 6.

**Fig. 8:**
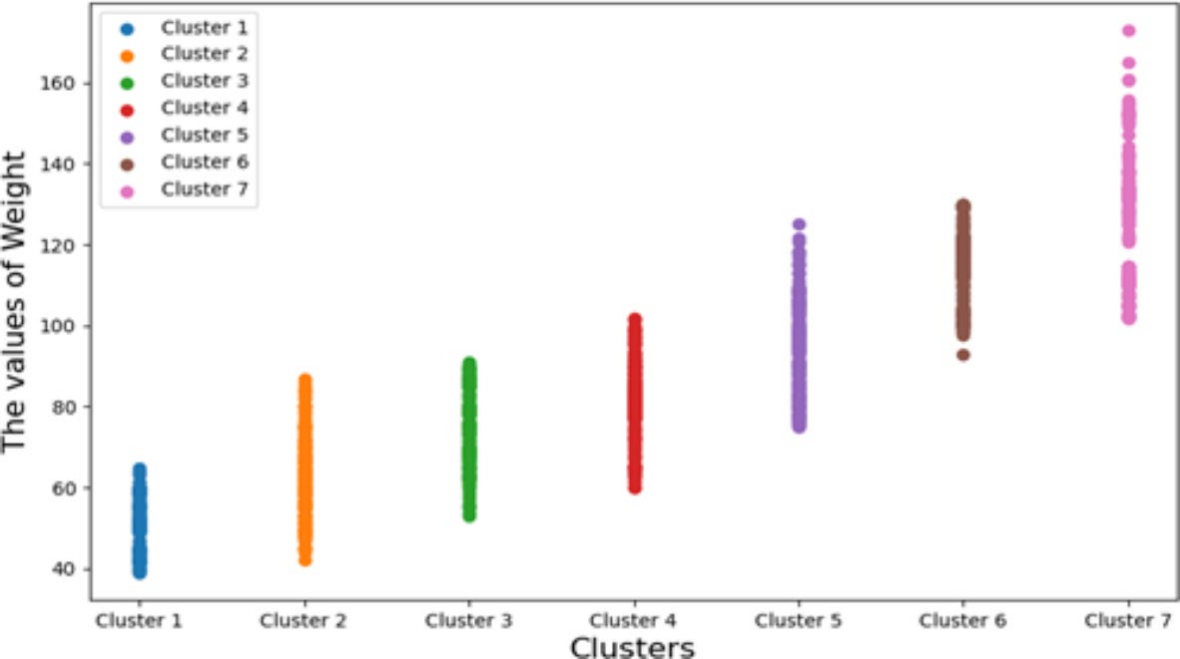
The obesity levels data are divided into 7 clusters using feature (Weight) through K-mean.

Among the 16 explanatory variables of the obesity levels dataset, since the feature (Weight) is closely related to the obesity level, the feature (Weight) is used to explain the seven clusters divided by K-means, as shown in Fig. 8. In Fig. 8, the horizontal axis is the seven clusters divided by K-means, and the vertical axis is the value of the feature (Weight). It can be found that the maximum value in the feature (Weight) falls in the cluster 7, and the minimum value in the feature (Weight) falls in the cluster 1.

**Fig. 9:**
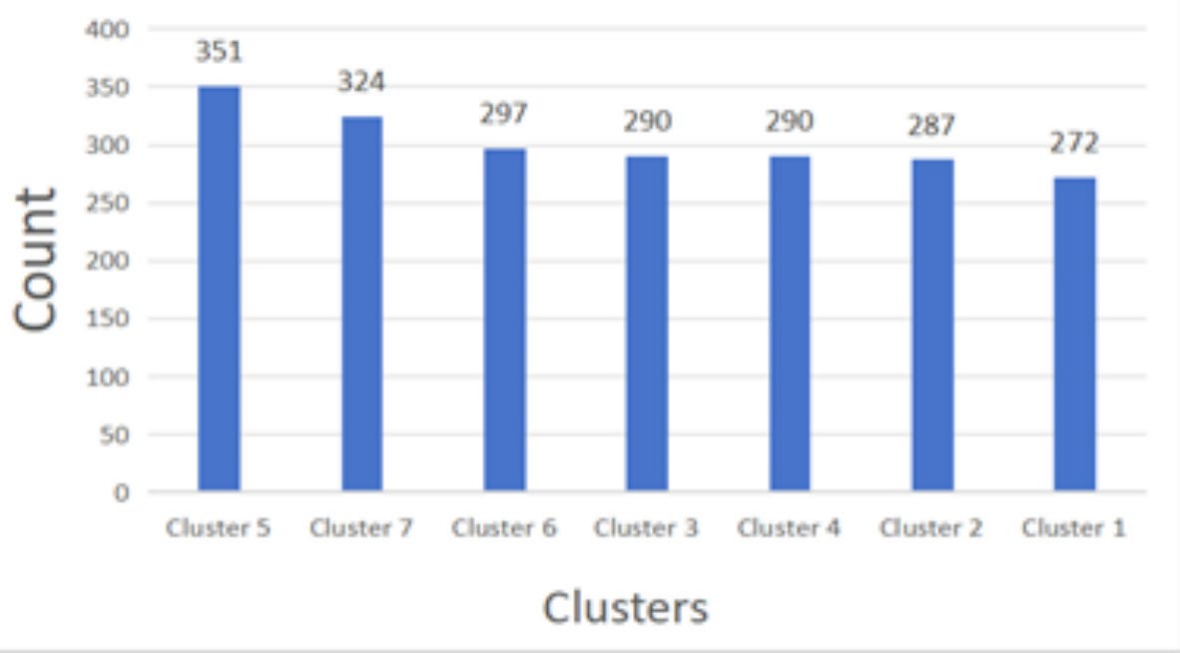
The clustering diagram of obesity levels dataset.

The obesity levels dataset has a total of 2,111 data. After K-means clustering, seven clusters are formed. The number of records in each cluster is presented from most to least, as shown in Fig. 9. From Fig. 9, we can find the cluster 5 has 351 records, which is the largest number of records among the seven clusters, which means that features with more similarities or commonalities are drawn in the cluster 5. The cluster 7 has 324 records, the cluster 6 has 297 records, the cluster 3 has 290 records, the cluster 4 has 290 records, the cluster 2 has 287 records, and the cluster 1 has 272 records, the least number among all the seven clusters, which means that the features with the least similarity or commonality are plotted in cluster 1.

### The analysis and discussion of the BLSMOTE + K-means + DT

**Fig. 10:**
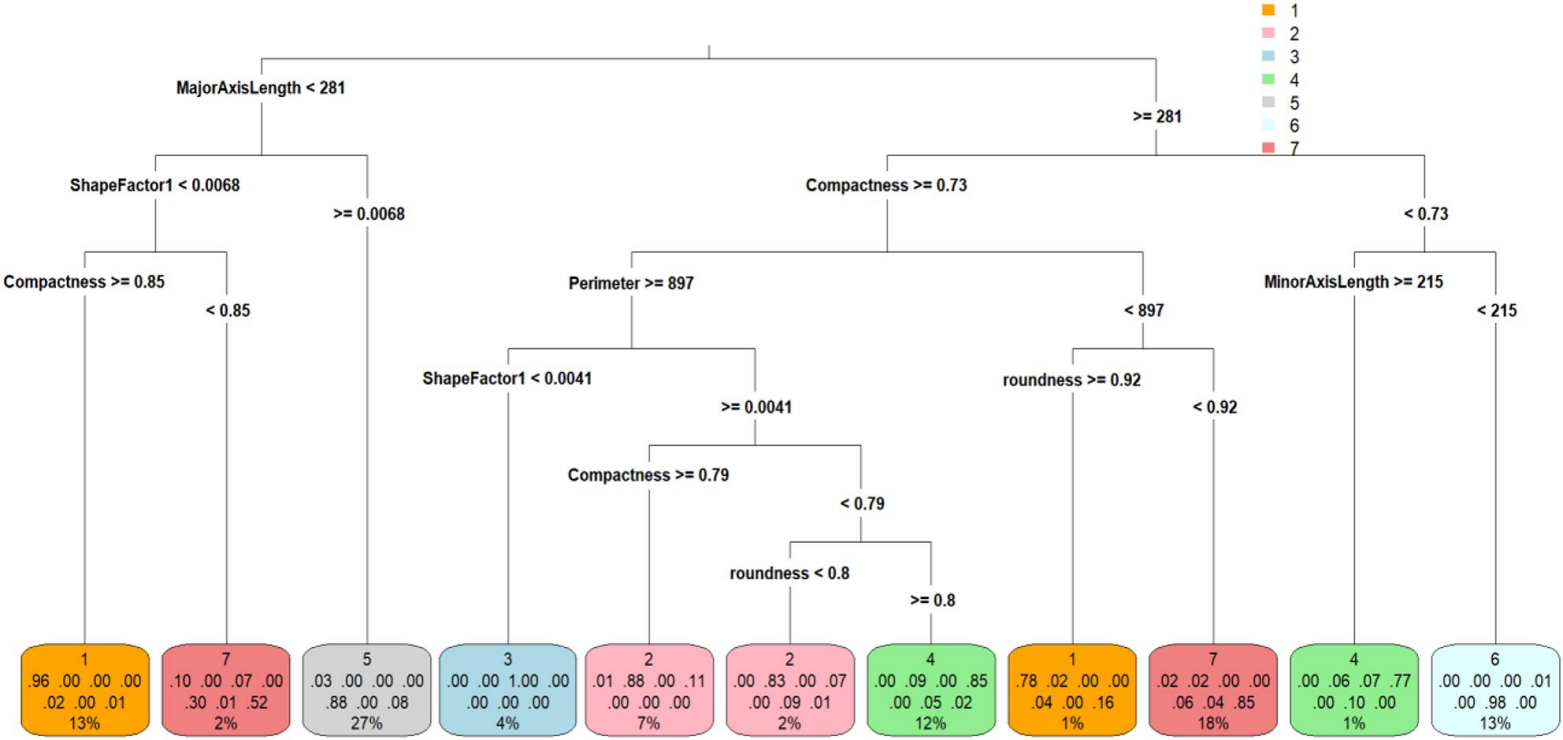
The BLSMOTE + K-means + DT training set decision diagram of dry bean dataset.

The training set classification accuracy of the BLSMOTE + K-means + DT is 89.98% for the dry bean dataset, which is better than 89.20% of only DT. The BLSMOTE + K-means + DTt training set decision diagram is shown in Fig. 10.

From Fig. 10, it can be found that when MajorAxisLength < 281, the left half tree is selected, and when MajorAxisLength $\:\ge\:$ 281, the right half tree is selected, and the BLSMOTE + K-means + DT decision tree has a total of eleven decision rules, as shown in Table 6.

**Table 6:** The eleven decision rules of the BLSMOTE + K-means + DT for dry bean dataset.

Rule	Explanation
1	When MajorAxisLength < 281, ShapeFactor1 < 0.0068, Compactness ≥ 0.85, the result is 1, which is Seker, and the number of samples accounts for 13%.
2	When MajorAxisLength < 281, ShapeFactor1 < 0.0068, and Compactness < 0.85, the result is 7, which is Dermason, and the number of samples accounts for 2%.
3	When MajorAxisLength < 281, ShapeFactor1 ≥ 0.0068, the result is 5, which is Horoz, and the number of samples accounts for 27%.
4	When MajorAxisLength ≥ 281, Compactness ≥ 0.73, Perimeter ≥ 897, ShapeFactor1 < 0.0041, the result is 3, which is Bombay, and the number of samples accounts for 4%.
5	When MajorAxisLength ≥ 281, Compactness ≥ 0.73, Perimeter **≥ **897, ShapeFactor1 ≥ 0.0041, Compactness ≥ 0.79, the result is 2, that is, Barbunya, and the number of samples accounts for 7%.
6	When MajorAxisLength ≥ 281, Compactness ≥ 0.73, Perimeter ≥ 897, ShapeFactor1 ≥ 0.0041, Compactness < 0.79, roundness < 0.8, the result is 2, that is, Barbunya, and the number of samples accounts for 2%.
7	When MajorAxisLength ≥ 281, Compactness ≥ 0.73, Perimeter ≥ 897, ShapeFactor1 ≥ 0.0041, Compactness < 0.79, roundness ≥ 0.8, the result is 4, which is Cali, and the number of samples accounts for 12%.
8	When MajorAxisLength ≥ 281, Compactness ≥ 0.73, Perimeter < 897, roundness ≥ 0.92, the result is 1, that is, Serker, and the number of samples accounts for 1%.
9	When MajorAxisLength ≥ 281, Compactness ≥ 0.73, Perimeter < 897, roundness < 0.92, the result is 7, which is Dermason, and the number of samples accounts for 18%.
10	When MajorAxisLength ≥ 281, Compactness < 0.73, and MinorAxisLength ≥ 215, the result is 4, which is Cali, and the number of samples accounts for 1%.
11	When MajorAxisLength ≥ 281, Compactness < 0.73, and MinorAxisLength < 215, the result is 6, which is Sira, and the number of samples accounts for 13%.

From the decision rules of BLSMOTE + K-means + DT in Table 6, it can be seen that the decision tree rules for Seker (the first type) are 1 and 8, the decision tree rules for Barbunya (the second type) are 5 and 6, the decision tree rules for Bombay (the third type) are only 4, the decision tree rules for Cali (the fourth type) are 7 and 10, the decision tree rules for Horoz (the fifth type) are only 3, the decision tree rules for Sira (the sixth type) are only 11, and the decision tree rules for Dermason (the seventh type) are 2 and 9.

**Fig. 11:**
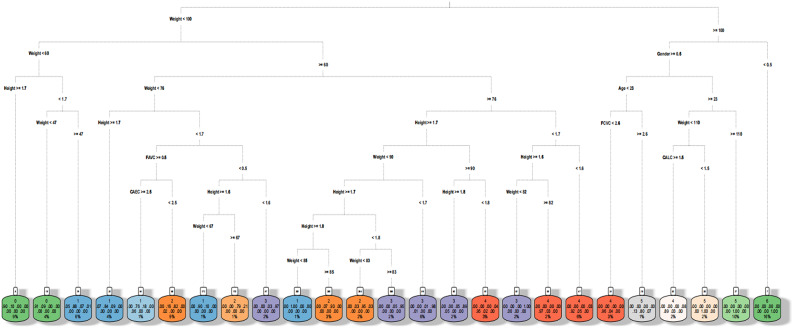
The BLSMOTE + K-means + DT training set decision diagram of obesity levels dataset.

The training set classification accuracy of the BLSMOTE + K-means + DT is 93.29% for the obesity levels dataset, which is better than 90.41% of only DT. The BLSMOTE + K-means + DTt training set decision diagram is shown in Fig. 11.

It can be found from Fig. 9 that weight is the root node, and the value 100 is the split point. When Weight < 100, the left half of the tree is selected, and when Weight ≥ 100, the right half of the tree is selected, and then the leaf nodes are recursed. In Fig. 11, the target variable content values ​​ (Insufficient_Weight, Normal_Weight, Overweight_Level_I, Overweight_Level_II, Obesity_Type_I, Obesity_Type_II, Obesity_Type_III) are replaced with 0 to 6 respectively using data transformation. The decision tree has a total of twenty-five decision rules, as shown in Table 7.

**Table 7:** The twenty five decision rules of the BLSMOTE + K-means + DT for obesity levels dataset.

Rule	Explanation
1	When Weight < 100, Weight < 60, Height ≥ 1.7, the result is 0, which means the population is underweight (Insufficient_Weight), and the sample size accounts for 9%.
2	When Weight < 100, Weight < 60, Height < 1.7, and Weight < 47, the result is 0, which means the population is underweight (Insufficient_Weight), and the sample size accounts for 4%.
3	When Weight < 100, Weigh t < 60, Height < 1.7, and Weight ≥ 47, the result is 1, which means the population is of normal weight (Normal Weight), and the sample size accounts for 6%.
4	When Weight < 100, Weight ≥ 60, Weight < 76, and Height ≥ 1.7, the result is 1, which means the population is of normal weight (Normal Weight), and the sample size accounts for 4%.
5	When Weight < 100, Weight ≥ 60, Weight < 76, Height < 1.7, FAVC ≥ 0.5, CAEC ≥ 2.5, the result is 1, which means the population is of normal weight (Normal_Weight), and the sample size accounts for 1%.
6	When Weight < 100, Weight ≥ 60, Weight < 76, Height < 1.7, FAVC ≥ 0.5, and CAEC < 2.5, the result is 2, which means the overweight level I (Overweight_Level_I) population, and the sample size accounts for 9%.
7	When Weight < 100, Weight ≥ 60, Weight < 76, Height < 1.7, FAVC < 0.5, Height ≥ 1.6, Weight < 67, the result is 1, which means the population is of normal weight (Normal_Weight), and the sample size accounts for 1%.
8	When Weight < 100, Weight ≥ 60, Weight < 76, Height < 1.7, FAVC < 0.5, Height ≥ 1.6, Weight ≥ 67, the result is 2, which means the population is Overweight Level I (Overweight_Level_I), and the sample size accounts for 1%
9	When Weight < 100, Weight ≥ 60, Weight < 76, Height < 1.7, FAVC < 0.5, Height < 1.6, the result is 3, which means the overweight level II (Overweight_Level_II) population, and the sample size accounts for 2%.
10	When Weight < 100, Weight ≥ 60, Weight ≥ 76, Height ≥ 1.7, Weight < 90, Height ≥ 1.7, Height ≥ 1.8, Weight < 85, the result is 1, which means the population is of normal weight (Normal_Weight), and the sample size accounts for 1%.
11	When Weight < 100, Weight ≥ 60, Weight ≥ 76, Height ≥ 1.7, Weight < 90, Height ≥ 1.7, Height ≥ 1.8, Weight ≥ 85, the result is 2, which means the overweight level I (Overweight_Level_I) population, and the sample number accounts for 3%.
12	When Weight < 100, Weight ≥ 60, Weight ≥ 76, Height ≥ 1.7, Weight < 90, Height ≥ 1.7, Height < 1.8, Weight < 83, the result is 2, which means the overweight level I (Overweight_Level_I) population, and the sample number accounts for 2%.
13	When Weight < 100, Weight ≥ 60, Weight ≥ 76, Height ≥ 1.7, Weight < 90, Height ≥ 1.7, Height < 1.8, Weight ≥ 83, the result is 3, which means the overweight level II (Overweight_Level_II) population, and the sample number accounts for 2%.
14	When Weight < 100, Weight ≥ 60, Weight ≥ 76, Height ≥ 1.7, Weight < 90, Height < 1.7, the result is 3, which means the overweight level II (Overweight_Level_II) population, and the sample size accounts for 6%.
15	When Weight < 100, Weight ≥ 60, Weight ≥ 76, Height ≥ 1.7, Weight ≥ 90, Height ≥ 1.8, the result is 3, which means the overweight level II (Overweight_Level_II) population, and the sample size accounts for 2%.
16	When Weight < 100, Weight ≥ 60, Weight ≥ 76, Height ≥ 1.7, Weight ≥ 90, Height < 1.8, the result is 4, which means the obesity level I (Obesity_Type_I) population, and the sample size accounts for 3%.
17	When Weight < 100, Weight ≥ 60, Weight ≥ 76, Height < 1.7, Height ≥ 1.6, Weight < 82, the result is 3, which means the overweight level II (Overweight_Level_II) population, and the sample size accounts for 2%.
18	When Weight < 100, Weight ≥ 60, Weight ≥ 76, Height < 1.7, Height ≥ 1.6, Weight ≥ 82, the result is 4, which means the obesity level I (Obesity_Type_I) population, and the sample size accounts for 2%.
19	When Weight < 100, Weight ≥ 60, Weight ≥ 76, Height < 1.7, Height < 1.6, the result is 4, which means the obesity level I (Obesity_Type_I) population, and the sample size accounts for 6%.
20	When Weight ≥ 100, Gender ≥ 0.5, Age < 23, FCVC < 2.6, the result is 4, which means the obesity type I (Obesity_Type_I) population, and the sample size accounts for 3%.
21	When Weight ≥ 100, Gender ≥ 0.5, Age < 23, and FCVC ≥ 2.6, the result is 5, which means the obesity type II (Obesity_Type_II) population, and the sample size accounts for 1%.
22	When Weight ≥ 100, Gender ≥ 0.5, Age ≥ 23, Weight < 110, CALA ≥ 1.5, the result is 4, which means the obesity type I (Obesity_Type_I) population, and the sample size accounts for 2%.
23	When Weight ≥ 100, Gender ≥ 0.5, Age ≥ 23, Weight < 110, CALA < 1.5, the result is 5, which means the obesity type II (Obesity_Type_II) population, and the sample size accounts for 2%.
24	When Weight ≥ 100, Gender ≥ 0.5, Age ≥ 23, and Weight ≥ 110, the result is 5, which means the obesity type II (Obesity_Type_II) population, and the sample size accounts for 10%.
25	When Weight ≥ 100 and Gender < 0.5, the result is 6, which means the obesity level III (Obesity_Type_III) population, accounting for 16% of the sample size.

It can be found from Table 7 that the decision rules for Insufficient_Weight (the first type) are 1 and 2, and the decision rules for Normal_Weight (the second type) are 3, 4, 5, 7 and 10. The decision rules that result in Overweight_Level_I (the third type) are 6, 8, 11, and 12, and the decision rules that result in Overweight_Level_II (the fourth type) are 9, 13, 14, 15, and 17. The decision rules that result in Obesity_Type_I (the fifth type) are 16, 18, 19, 20, and 22, and the decision rules that result in Obesity_Type_II (the sixth type) are 21, 23, and 24. The result is that the decision rule for Obesity_Type_III (the seventh type) is only 25.

### The analysis and discussion of the BLSMOTE + K-means + RF

**Fig. 12:**
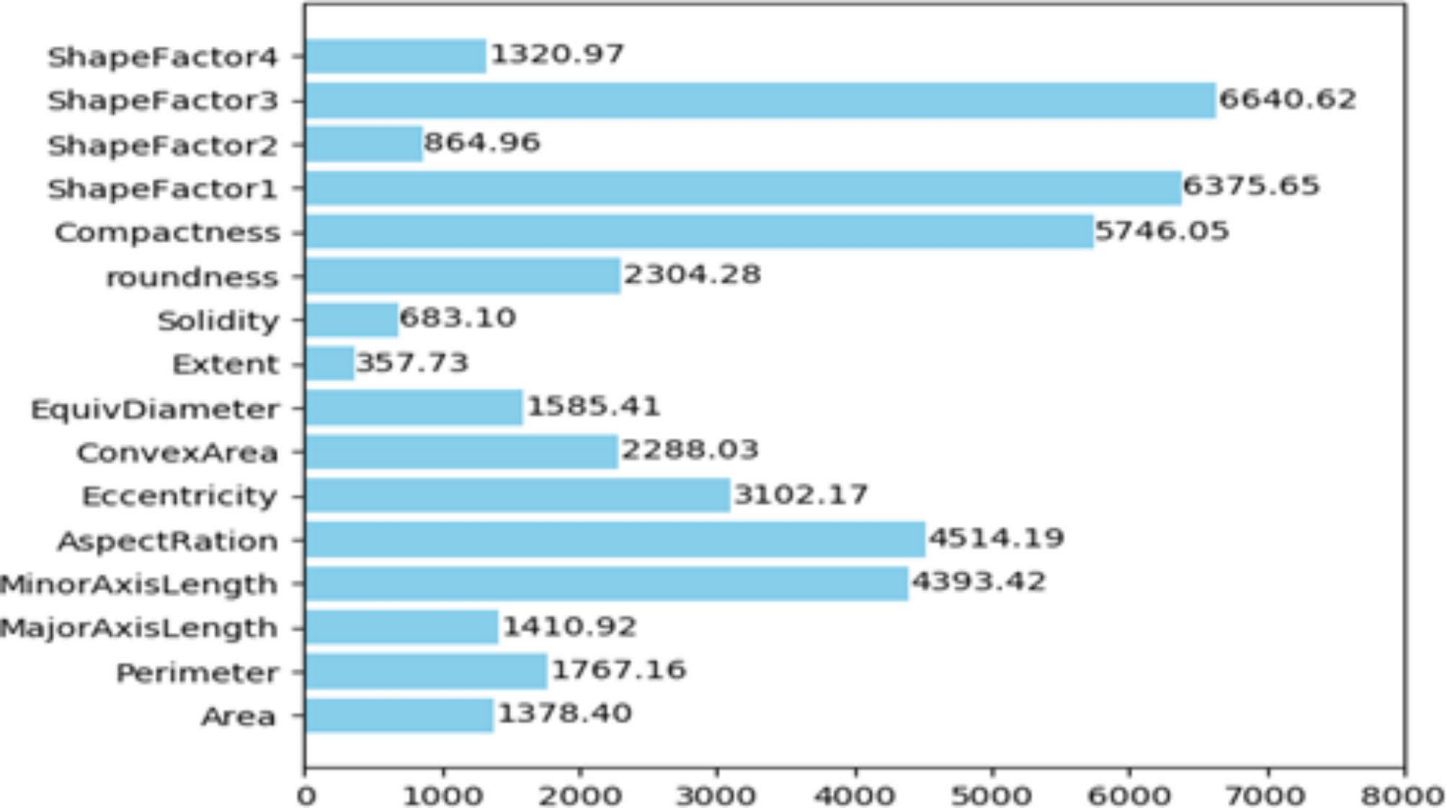
The average impurity reduction value of dry bean features of the BLSMOTE + K-means + RF.

**Fig. 13:**
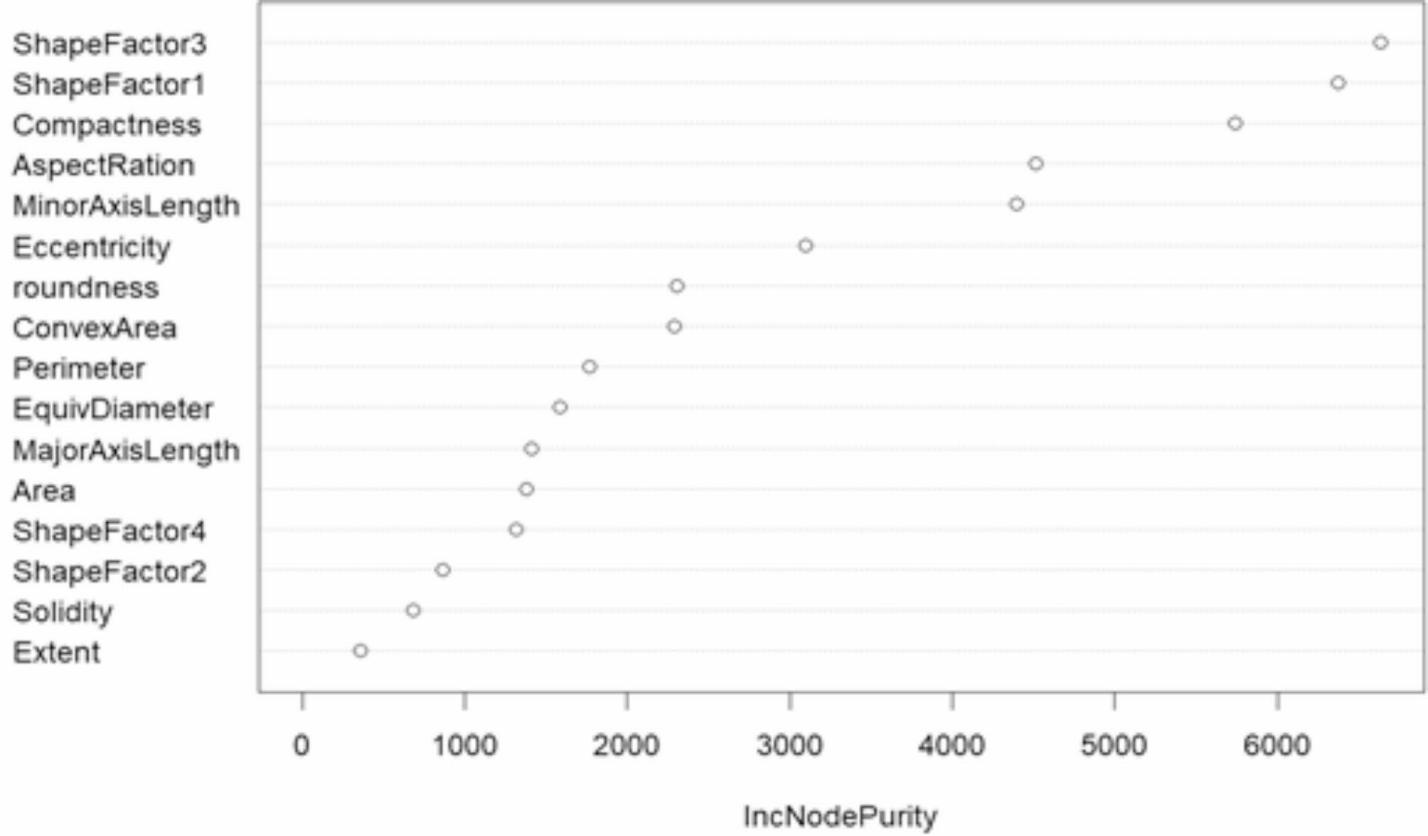
The BLSMOTE + K-means + RF feature importance ranking diagram of dry bean.

The dry bean dataset in the study of BLSMOTE + K-means + RF, ntree was set to 100, and the training set classification accuracy of the BLSMOTE + K-means + RF is 92.58% for the dry bean dataset, which is better than 92.46% of only RF. The importance ranking of dry bean features is based on the average impurity reduction as the indicator, which is the weighted average of the Gini impurity indicator reduction within the random forest range. The larger the indicator, the more important the attribute is. The average impurity reduction value of dry bean features by BLSMOTE + K-means + RF is shown in Fig. 12. The dry bean feature importance ranking of BLSMOTE + K-means + RF is given, as shown in Fig. 13.

As shown in Figs. 12 and 13, it can be found that the order of importance of dry bean features is: ShapeFactor3 > ShapeFactor1 > Compactness > AspectRation > MinorAxisLength > Eccentricity > roundness > ConvexArea > Perimeter > EquivDiameter > MajorAxisLength > Area > ShapeFactor4 > ShapeFactor2 > Solidity > Extent. Among them, ShapeFactor3 has the greatest impact on the dry bean classification results. The main reason is that the appearance shapes of different dry beans vary greatly, and it is easier to determine the category of dry beans through the ShapeFactor3 feature.

**Fig. 14:**
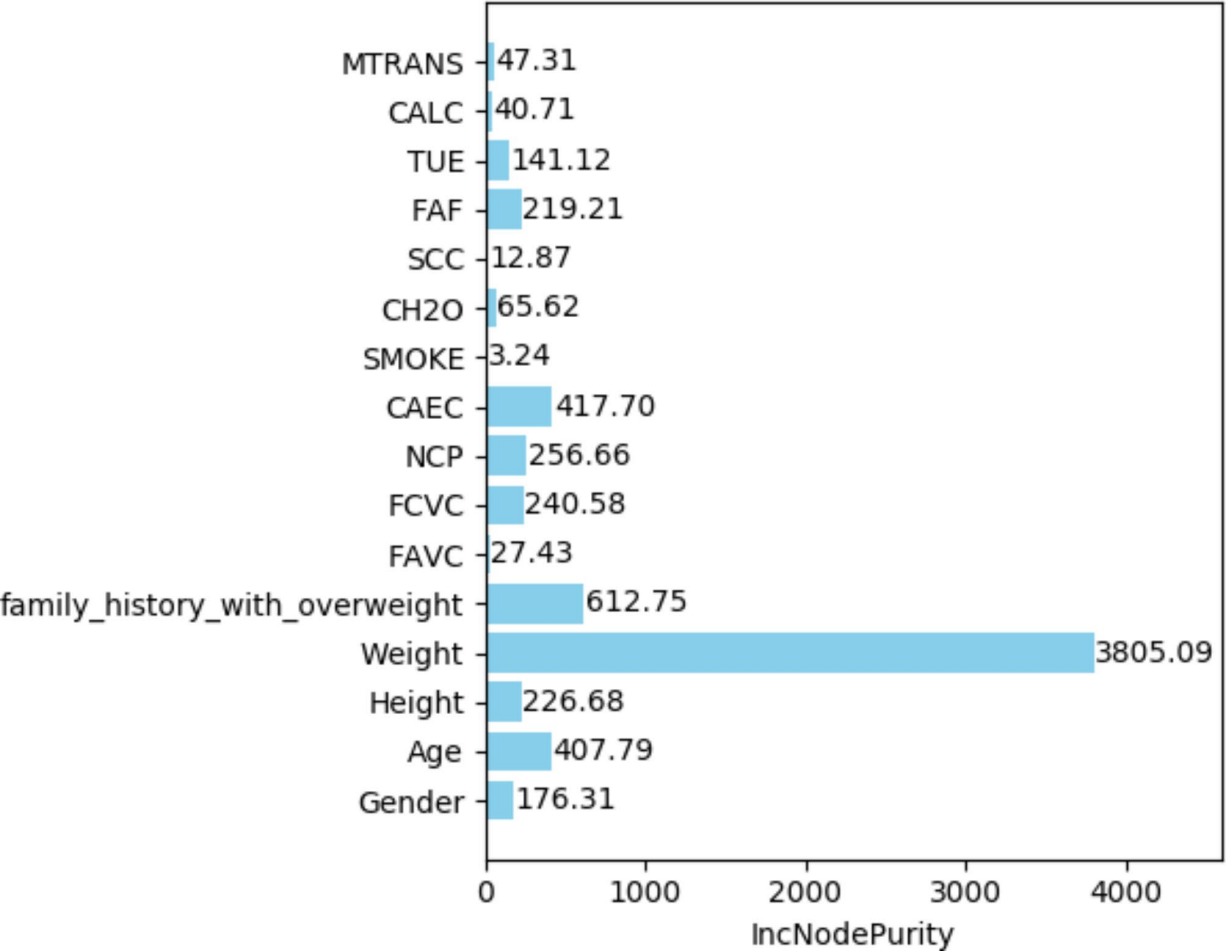
The average impurity reduction value of obesity levels features of the BLSMOTE +K-means + RF.

**Fig. 15:**
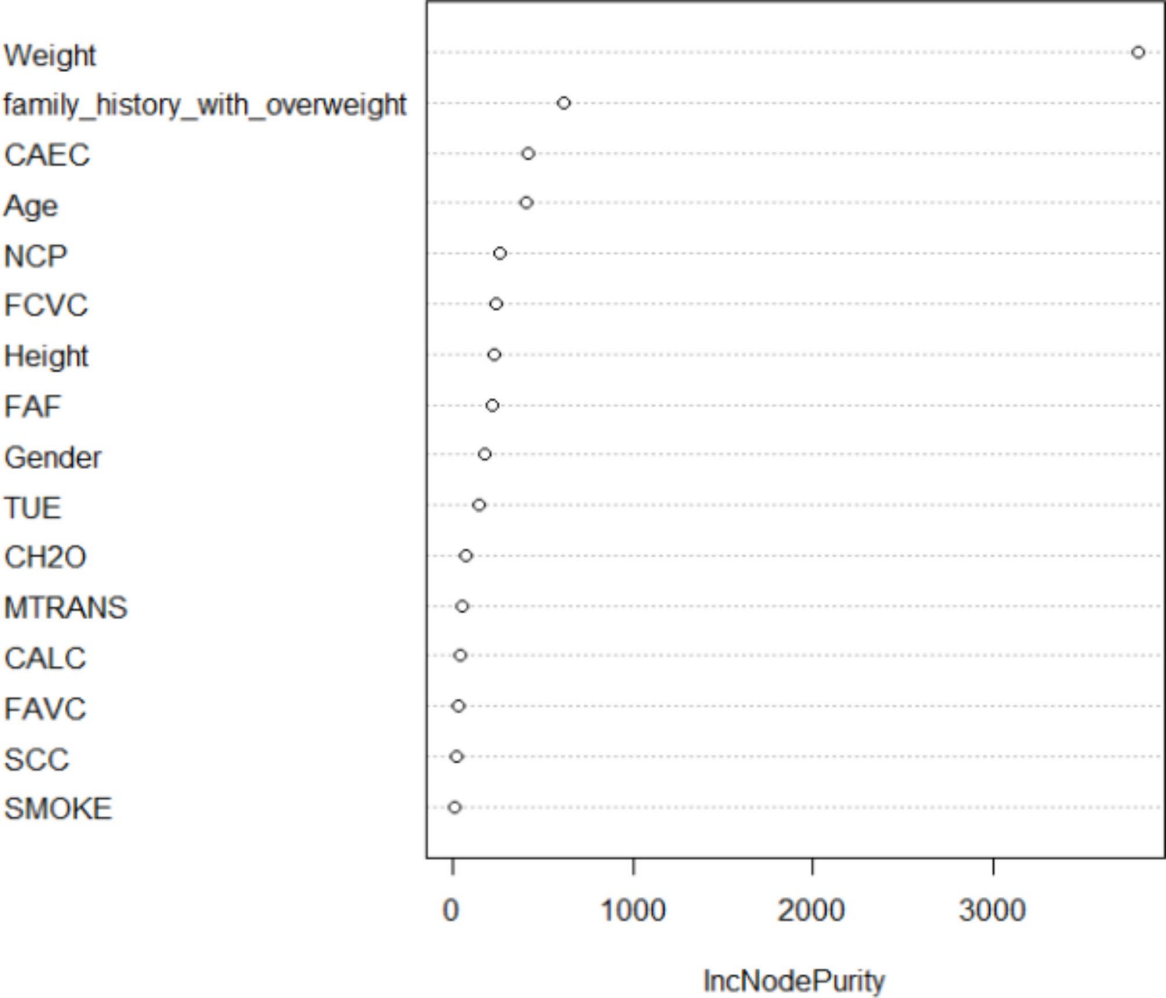
The BLSMOTE + K-means + RF feature importance ranking diagram of obesity levels dataset.

The obesity levels dataset in the study of BLSMOTE + K-means + RF, ntree was set to 100, and the training set classification accuracy of the BLSMOTE + K-means + RF is 99.13% for the obesity levels dataset, which is better than 97.99% of only RF. The importance ranking of obesity levels features is based on the average impurity reduction as the indicator, which is the weighted average of the Gini impurity indicator reduction within the random forest range. The larger the indicator, the more important the attribute is. The average impurity reduction of obesity levels features by BLSMOTE + K-means + RF is shown in Fig. 14. The obesity levels feature importance ranking of BLSMOTE + K-means + RF is given, as shown in Fig. 15.

As shown in Figs. 14 and 15, it can be found that the order of importance of obesity levels features is: Weight >family_history_with_over > Age > CAEC > FCVC > Height > NCP > Gender > FAF > TUE > MTRANS > CH2O > CALC > FAVC > SCC > SMOKE. Among them, the feature (Weight) has the greatest impact on the dry bean classification results.

## Conclusions

This study combined BLSMOTE + K-means with machine learning, namely BLSMOTE + K-means + SVM, BLSMOTE + K-means + DT, and BLSMOTE + K-means + RF, to eslish prediction models for dry bean datasets and obesity level datasets to improve the classification performance of traditional machine learning methods. The training set classification accuracy of BLSMOTE + K-means + SVM on the dry bean dataset is 98.86%, which is better than the 94.01% of only SVM. The training set classification accuracy of BLSMOTE + K-means + SVM on the obesity level dataset is 99.57%, which is better than the 98.99% of only SVM. The training set classification accuracy of BLSMOTE + K-means + RF on the dry bean dataset is 92.58%, which is better than the 92.46% of only RF. The training set classification accuracy of BLSMOTE + K-means + RF on the obesity level dataset is 99.13%, which is better than the 97.99% of only RF. The training set classification accuracy of BLSMOTE + K-means + DT on the dry bean dataset is 89.98%, which is better than the 89.20% of only DT. The training set classification accuracy of BLSMOTE + K-means + DT on the obesity level dataset is 93.29%, which is better than the 90.41% of only DT. In addition, the precision, recall, f1-score, and AUC performance indicators of BLSMOTE + K-means + SVM on the dry bean dataset are 0.9736, 0.9743, 09739, and 0.9831, respectively, which are better than 0.9402, 0.9437, 0.9419, and 0.9527 of only SVM. In addition to precision, the recall, f1-score, and AUC performance indicators of BLSMOTE + K-means + RF on the dry bean dataset are 0.9183, 0.9091, and 0.9277, respectively, which are better than 0.9071, 0.9062 and 0.9245, of only RF. The precision, recall, f1-score, and AUC performance indicators of BLSMOTE + K-means + DT on the dry bean dataset are 0.9098, 0.9069, 0.9083, and 0.9102, respectively, which are better than 0.9042, 0.8959, 0.9000 and 0.9088 of only DT. The precision, recall, f1-score, and AUC performance indicators of BLSMOTE + K-means + SVM on the obesity level dataset are 0.9736, 0.9743, 0.9739, and 0.9885, respectively, which are better than 0.9402, 0.9437, 0.9419, and 0.9807 of only SVM. The precision, recall, f1-score, and AUC performance indicators of BLSMOTE + K-means + RF on the obesity level dataset are 0.9046, 0.9256, 0.9150, and 0.9835, respectively, which are better than 0.8668, 0.8754, 0.8711, and 0.9747 of only RF. The precision, recall, f1- score, and AUC performance indicators of BLSMOTE + K-means + DT on the obesity level dataset are 0.8060, 0.8765, 0.8398, and 0.9642, respectively, which are better than 0.8052, 0.8151, 0.8101, and 0.9312 of only DT. The experimental results show that the BLSMOTE + K-means + SVM, BLSMOTE + K-means + RF, and BLSMOTE + K-means + DT proposed in this study have indeed improved the traditional only SVM, only RF, only DT in classification accuracy and precision, recall, f1-score, and AUC performance indicators. Because the imbalanced data is first processed by the BLSMOTE, its advantage is that it uses samples on the boundary of minority class samples to generate new samples, which can reduce the impact of noise on model building; and the advantage of K-means clustering is that it can divide data into different groups based on similarities or common features.

In this study, the BLSMOTE + K-means + DT generated eleven decision rules for the dry bean dataset and twenty-five decision rules for the obesity levels dataset. BLSMOTE + K-means + RF also gave the importance ranking of the explanatory variables for the above two datasets. From the experimental results, the performance of the proposed algorithm can indeed effectively improve the traditional machine learning method. The following suggestions are made for future work:

(1) In future work, we can consider using intelligent algorithms to optimize the parameters of SVM to improve classification accuracy.

(2) In the future, we can use Adaboost, and XGB as a comparison with the BLSMOTE + K-means + SVM, BLSMOTE + K-means + DT, and BLSMOTE + K-means + RF proposed in this paper, which is believed to enrich the content of this study.

## Data Availability

All data generated or analyzed during this study are included in this published paper.
